# Heparanase activity in alveolar and embryonal rhabdomyosarcoma: implications for tumor invasion

**DOI:** 10.1186/1471-2407-9-304

**Published:** 2009-08-28

**Authors:** Valentina Masola, Claudio Maran, Evelyne Tassone, Angelica Zin, Angelo Rosolen, Maurizio Onisto

**Affiliations:** 1 Department of Experimental Biomedical Sciences, University of Padova, Viale G. Colombo 3, 35121 Padova, Italy; 2 Division of Hematology-Oncology, Department of Paediatrics, University-Hospital of Padova, Via Giustiniani 3, 35128 Padova, Italy

## Abstract

**Background:**

Rhabdomyosarcoma (RMS) is a malignant soft tissue sarcoma of childhood including two major histological subtypes, alveolar (ARMS) and embryonal (ERMS) RMS. Like other human malignancies RMS possesses high metastatic potential, more pronounced in ARMS than in ERMS. This feature is influenced by several biological molecules, including soluble factors secreted by tumor cells, such as heparanase (HPSE). HPSE is an endo-β-D-glucuronidase that cleaves heparan sulphate proteoglycans.

**Methods:**

We determined HPSE expression by Western blot analysis in ARMS and ERMS cells lines and activity in supernatants by an ELISA assay. Stable *HPSE *silencing has been performed by shRNA technique in RH30 and RD cell lines and their invasiveness has been evaluated by Matrigel-invasion assay. HPSE activity and mRNA expression have also been quantified in plasma and biopsies from RMS patients.

**Results:**

HPSE expression and activity have been detected in all RMS cell lines. Stable *HPSE *silencing by shRNA technique determined a significant knockdown of gene expression equal to 76% and 58% in RH30 and RD cell lines respectively and induced a less invasive behaviour compared to untreated cells. Finally, we observed that *HPSE *mRNA expression in biopsies was higher than in foetal skeletal muscle and that plasma from RMS patients displayed significantly more elevated HPSE levels than healthy subjects with a trend to higher levels in ARMS.

**Conclusion:**

In conclusion, our data demonstrate for the first time HPSE expression and activity in RMS and highlight its involvement in tumor cell invasion as revealed by shRNA silencing. Moreover, HPSE expression in RMS patients is significantly higher with respect to healthy subjects. Further studies are warranted to assess possible relationships between HPSE and clinical behaviour in RMS.

## Background

Rhabdomyosarcoma (RMS) is the most common soft-tissue cancer occurring in childhood. It originates from primitive mesenchymal cells committed to myogenic differentiation. RMS belongs to the broader category of small blue round cell tumors of childhood and histologically resembles normal foetal skeletal muscle [1]. It can be classified into two main histological subtypes with distinct appearances and clinical behaviours. The rarer alveolar type (ARMS) arises mainly in the extremities and trunk. It is also associated with worse prognosis and higher disease stage at diagnosis. The unfavourable prognosis is related to the propensity for early and wide dissemination, often involving the lungs, bone marrow and bones and poor response to chemotherapy. In contrast, the embryonal rhabdomyosarcoma (ERMS) usually affects younger children, at more favourable sites, and shows a less aggressive clinical behaviour than ARMS [2,3].

The frequent tumor dissemination and metastasis characteristics of ARMS, less common in ERMS, suggest a different expression of molecules involved in these events between the two subtypes. An important process in host tissue invasion is the extracellular matrix (ECM) degradation due to secreted and cell surface bound protease and glycosidase activities. These ECM-remodelling enzymes affect and modify cell and tissue functions [4]. The ECM is composed of a complex network of macromolecules which fills the extracellular space in tissues and provides an essential physical barrier among cells, as well as a molecular scaffold for cell growth, migration, differentiation and survival. It also undergoes continuous remodelling during development and in a variety of pathological conditions such as cancer. While intensive research focused on enzymes capable of degrading protein components in the ECM has been established [5,6], presently attention is directed towards enzymes (e.g., heparanase) cleaving glycosaminoglycan side chains. The heparan sulphate proteoglycans (HSPGs), the main polysaccharide component of the ECM, are ubiquitous macromolecules associated with the cell surface and the ECM of a wide range of cells in vertebrate and invertebrate tissues. Heparan sulphate (HS) plays a key role in the self-assembly and integrity of the ECM. In fact, HS chains can bind large number of proteins and several bioactive molecules like growth factors, chemokines, cytokines and enzymes to the cell surface and ECM, thereby regulating their availability and function in the control of some physiological processes, such as cell-cell and cell-ECM interactions [7]. Malignant tumor growth, neo-vascularization and metastasis represent pathological invasive phenomena that involve the enzymatic degradation of the ECM. Heparanase (HPSE) is an endo-β-D-glucuronidase that cleaves HS side chains of HSPGs. Heparanase cleavage of HS in the ECM, particularly in epithelial and sub-endothelial basement membranes, is a critical step in cancer development and progression in correlation with metastatic potential, tumor vascularity and reduced survival of cancer patients [8]. Its activity is regulated by gene expression and pro-enzyme activation. In fact heparanase is synthesized as a 65 kDa inactive precursor whose activation involves proteolytic cleavage, resulting in 8 and 50 kDa protein subunits that heterodimerize to form the active enzyme. Remarkably, the active form of heparanase is secreted in the extracellular environment [9,10]. Recent studies have shown that this enzyme is upregulated in an increasing number of primary human tumors providing a strong clinical support for its pro-metastatic and pro-angiogenic features [11,12], thus making it a promising target for the development of anti-cancer treatments [13,14].

In contrast to frequent cancers occurring in adults, little information is available on RMS with regard to molecules involved in tumor invasion. We analyzed HPSE expression and activity in ARMS and ERMS cell lines and investigated the relationships of the different metastatic phenotype of ARMS and ERMS with expression and activity of heparanase.

## Methods

### Cell cultures

Human alveolar rhabdomyosarcoma (ARMS) cell lines RH30, RH4, RH18, RH28 and human embryonal rhabdomyosarcoma (ERMS) cell lines RD, SMS-CTR, RH36, CCA were grown in RPMI (EuroClone, Pavia, Italy) supplemented with 10% foetal bovine serum (Biochrom AG, Berlin, Germany), 2 mM L-glutamine, penicillin (100 U/ml) and streptomycin (100 μg/ml). All cell lines were maintained at 37°C in a 5% CO_2 _water-saturated atmosphere.

### Plasma and tumor RNA collection from RMS patients

A total of 3 ml of peripheral blood was obtained at the time of diagnosis in sodium citrate [15]. All plasma samples were aliquoted, frozen at -80°C until analysis and thawed once.

The plasma study cohort consisted of 15 paediatric patients (5 with ARMS, 10 with ERMS) and 10 healthy subjects as control. In 12 out of 15 RMS cases (3 ARMS, 9 ERMS), tumor tissue was available for RNA purification. For RMS patients, the mean age was 6.3 years (range 1 – 15 yrs) with 8 girls and 7 boys at the time of diagnosis while, for healthy subjects, the mean age was 8.9 years (range 2 – 13 yrs) with 3 girls and 7 boys. Informed parental consent was obtained in each case.

### RNA extraction and cDNA synthesis

Total RNA was extracted from cell monolayers using the "GenElute Mammalian Total RNA Miniprep" Kit (Sigma-Aldrich, Milan, Italy) including DNase treatment (DNASE70, Sigma), according to manufacturer's instructions.

Tumor RNA was isolated by using RNA-zol (Tel-Test, Friendswood, USA) following the manufacturer's instructions.

Yield and purity were checked by Nanodrop (EuroClone).

1 μg of total RNA from each sample was reverse transcribed into cDNA using 500 ng random primers and 200 U SuperScript II Reverse Transcriptase (Invitrogen, Milan, Italy).

### Cloning of HPSE and GAPDH to obtain an internal standard

Total RNA from RH30 cells was reverse transcribed and subsequently subjected to PCR amplification by using *HPSE *and *GAPDH *specific primers (Table 1). The PCR products (136 bp for *HPSE *and 112 bp for *GAPDH*) were electrophoresed on a 2% agarose gel in 1× TAE buffer [50 mM Tris-HCl (pH 8.0), 20 mM sodium acetate, 2 mM Na_2_EDTA] and visualized by ethidium bromide staining. After isolation they were purified with the "QIAquickTM Gel Extraction" Kit (Qiagen, Milan, Italy) and subsequently cloned directly into pCR^®^II-TOPO^® ^Vector (TOPO TA Cloning^® ^system, Invitrogen). They were then sequenced on both strands using Big Dye terminator v3.1 protocol on ABI PRISM 310 Genetic Analyzer (Applied Biosystems, Milan, Italy).

**Table 1:** primers sequences

Gene	Sequences 5'→ 3'
HPSE	d – ATTTGAATGGACGGACTGC
	r – GTTTCTCCTAACCAGACCTTC
MMP2	d – GCGGCGGTCACAGCTACTT
	r – CACGCTCTTCAGACTTTGGTTCT
MMP9	d – CCTGGAGACCTGAGAACCAATC
	r – CCACCCGAGTGTAACCATAGC
MMP14	d – TGCCATGCAGAAGTTTTACGG
	r – TCCTTCGAACATTGGCCTTG
GAPDH	d – ACACCCACTCCTCCACCTTT
	r – TCCACCACCCTGTTGCTGTA

### Quantitative Real-time PCR

We performed a quantitative Real-time PCR assay based on the use of SYBR Green I (Applied Biosystems) as fluorescent intercalation dye into double-stranded DNA during the amplification cycles. The assays were performed in 96 multi-well PCR plates covered with optical tapes in the Applied Biosystems 7500 Real-time PCR System in a final volume of 25 μl, containing 10 ng of cDNA, 12.5 μl of Power Master Mix 2X (Applied Biosystems), 5 μmoles of either *HPSE*, Matrix Metalloproteinases (*MMPs*) or *GAPDH *forward and reverse primers, and water. The specific primer sequences are listed in Table 1.

The reaction was subjected to denaturation at 95°C for 10 minutes followed by 40 cycles of denaturation at 95°C for 30 seconds, annealing and elongation at 60°C for 1 minute.*HPSE *mRNA expression levels were evaluated by an absolute quantitative Real-time PCR in RMS cell lines and biopsies from RMS patients. Plasmids containing *HPSE *and *GAPDH *fragments were used as templates to obtain the standard curves. The relative *HPSE *mRNA expression levels were described as *HPSE *copy number/*GAPDH *copy number ratios. After the amplification, the software directly defined the standard curve of the experiment based on the Ct for each gene in analysis. In this way, a linear regression line was plotted and the resulting equation was used to calculate the copy number for the unknown samples [16].*MMPs *mRNA expression levels were evaluated by a relative quantitative Real-time PCR in RMS cell lines and normalized to *GAPDH*. The comparative Ct method (ΔΔCt) was used to quantify *MMPs *gene expression and the relative quantification (RQ) was calculated as 2^-ΔΔCt^. *MMP2 *expression level in RH30 wild type cell line was regarded as 100% and *MMP2*, *MMP9 *and *MMP14 *levels in the other samples were calculated relative to this value.

### Western blot

Sub-confluent cells were washed with PBS, lysed and scraped in lysis buffer [50 mM Tris-HCl (pH 5.0), 150 mM NaCl, 0.01% Triton X-100] with Protease Inhibitor Cocktail "Complete" (Roche, Milan, Italy). Cellular lysates were sonicated, centrifuged at 4°C for 30 minutes at 15,000 × g and finally quantified using Bio-Rad Protein Assay (Bio-Rad, Milan, Italy). Total proteins (50 μg) and platelet extract (5 μg) used as positive control were treated in reducing sample buffer [2% SDS, 80 mM Tris-HCl (pH 6.8), 10% glycerol, 0.005% bromophenol blue, 10% β-mercaptoethanol] and boiled for 10 minutes. Subsequently the samples were resolved in 9% SDS-PAGE and electro-transferred for 2.5 hours at 4°C to nitrocellulose membranes. Membranes were exposed to primary polyclonal antibody (sc-25825, Santa Cruz Biotechnolgy, Santa Cruz, CA). After three washes in TBST [20 mM Tris-HCl (pH 7.4), 150 mM NaCl, 0.05% Tween-20], the membranes were incubated with a secondary peroxidase-conjugated antibody (sc-2004, Santa Cruz Biotechnology). The signal was detected by "SuperSignals West Pico Chemiluminescent" substrate solution (Pierce, Rockford, IL), according to the manufacturer's instructions.

### HPSE ELISA

The quantitative detection of HPSE in RMS cell culture supernatants was performed with "Heparanase assay" Kit from AMS Biotechnology (Milton Abingdon, Oxon, UK) according to the manufacturer's instructions. Briefly, the ELISA plate was rehydratated by adding 100 μl of PBS and incubated at room temperature for 30 minutes. After removing PBS, 50 μl of the reaction buffer and 50 μl of samples were added in each well. 50 μl of reaction buffer and 50 μl of platelet protein extract diluted 1:100 in 1× Heparanase buffer were used as negative and positive controls respectively. The samples were serum-free conditioned media obtained from the different cell lines. The micro-well plate was then incubated 1 hour at 37°C and then each well was washed four times with PBT [PBS + 0.1% v/v Tween-20] and subsequently twice with PBS. To each well was added100 μl of the supplied Strep-HRP complex and incubated 1 hour at room temperature on a plate shaker. The micro-well plate was washed five times with PBS and subsequently 100 μl of peroxidase substrate were added at room temperature. The plate was gently shaken and the absorbance determined at 450 nm in an ELISA plate reader. Activity of each sample was calculated as described by the manufacturer and represented as ng of HS removed per minute.

### Transfection of HPSE shRNA plasmid into RH30 and RD cell lines

To obtain stably *HPSE *silenced cell lines, we used four different shRNAs targeting human heparanase (NM_006665). The sequence of the 29 mer shRNAs are listed in Table 2.

**Table 2:** shRNA 29 mer sequences

shRNA	Sequences
pHPSE-1	TTATGTGGCTGGATAAATTGGGCCTGTCA
pHPSE-2	GTTCAAGAACAGCACCTACTCAAGAAGCT
pHPSE-3	GTGGTGATGAGGCAAGTATTCTTTGGAGC
pHPSE-4	TCGTTCCTGTCCGTCACCATTGACGCCAA

The *HPSE *gene-specific shRNA expression cassettes as well as the negative control shRNA pRS plasmid (TR20003) and the negative control shRNA pRS non-effective GFP plasmid (TR30003), were purchased from OriGene (Rockville, MD, USA). Transcribed shRNAs were reconstituted with 50 μl of RNase-Free water (Gibco, Invitrogen) to prepare a 100 ng/μl stock solution. RH30 and RD cell lines were seeded in 6-well plates at a density of 2.0 × 10^5 ^and 1.5 × 10^5 ^cells per well respectively. After 24 hours, cells at 70 – 80% confluence were transfected with 2 μg of each shRNA plasmid in serum-free medium using TransIT-LT1 transfection reagent (Mirus, Madison, WI) according to the manufacture's instruction. Control cells were treated with the same amount of TransIT-LT1 transfection reagent (Mock). 48 hours after transfection, RH30 and RD cells were placed for some weeks under selection with 0.5 and 1.0 μg/ml of puromycin (Sigma) respectively. From RH30 and RD cell lines, three different stable clones were selected for subsequent analysis.

### Proliferation assay

The proliferation of RH30 and RD cell lines was evaluated by MTT assay. For both cell lines, wild type and silenced cells were tested as well as the relative negative controls. Cells in mid-log phase were seeded in 96-well culture plate and cultured for 24, 48 and 72 hours in their medium. Subsequently 10 μl of 3-(4,5-dimethylthiazol-2-yl)-2,5-diphenyltetrazolium bromide (MTT) solution (5 mg/ml in phosphate-buffered saline) was added to each well, cells were incubated for 4 h at 37°C and the precipitates were dissolved in 150 μl of dimethyl sulfoxide. The number of proliferating cells was evaluated by measuring optical density at 540 nm on a microtiter plate reader.

### Invasion assay

The invasive behaviour of ARMS RH30 and ERMS RD cell lines was assessed by using the Boyden-chamber assay according to the method of Albini *et al*. [17], with slight modifications. For both cell lines, wild type and silenced cells were tested as well as the relative negative controls. The filters used in the assay were "Isopore Membrane Filters" (Millipore, Milan, Italy) with pore size of 8.0 μm. Filters were coated with 50 μl of Matrigel solution (BD Biosciences, Milan, Italy), an artificial basement membrane that contains abundant HSPGs, previously prepared at a concentration of 0.225 μg/μl. Tumor cells (2.0 × 10^5 ^cells/ml) resuspended in 800 μl serum-free medium were layered on top of the polymerized gel in the upper compartment of the chamber while, in the lower compartment, conditioned serum-free medium from NIH3T3 cell line was used as chemo-attractant. After 5-hour incubation at 37°C, Matrigel was removed, the filters were washed in water, fixed in 100% ethanol for 5 minutes and stained with 1% toluidine blue/1% sodium tetraborate for 2 minutes. After staining, the filters were let to dry and photographed using Canon PowerShot G6 camera. Images were analyzed by ImageJ software http://rsb.info.nih.gov/ij/.

### HPSE plasma assay

HPSE plasma activity was quantified according to the method of Xu *et al*. [18]. This assay is based on the ability of HPSE to degrade heparan-sulphate proteoglycans present in the Matrigel. 25 μl of Matrigel was dissolved in ice-cold PBS at a concentration of 200 μg/ml and used to coat ELISA plates and left to dry at room temperature for 1.5 hour. Samples were diluted at 1:4 in HPSE buffer [0.1 M Sodium Acetate (pH 5.0), 0.1 mg/ml BSA, 0.01% Triton X-100, Protease Inhibitor Cocktail]. The plates were washed once with PBT [PBS + 0.05% v/v Tween-20] and then samples were added and incubated overnight at 37°C. The wells were washed with PBT and blocked with the appropriate buffer [PBT, 0.5% BSA, 1 mM EDTA] at room temperature for 2 hours. After PBT washing, primary monoclonal antibody anti-HS (clone HepSS-1, Seikagaku, Tokyo, Japan) diluted 1:500 was added and incubated in blocking buffer at room temperature for 1 hour. After PBT washing, goat anti-mouse IgM-HRP secondary antibody (sc-2973, Santa Cruz Biotechnology) diluted 1:1000 was added and incubated at room temperature for 1 hour. Then 50 μl of the ABTS [2,2-azino-bis-(3-ethylbenzthiazoline-6-sulphonic acid), Sigma] was added to each well. Finally, the reaction was blocked with 50 μl per well of 1% SDS and the OD405 absorbance was read in an ELISA plate reader.

HPSE activity in each plasma sample was calculated as the difference between the OD405 value with or without heparin (Sigma) at the final concentration of 50 μg/ml. This inhibitor was used to ensure the detection of the specific HPSE activity in plasma samples.

### Statistical analysis

Differences in HPSE plasma levels and *HPSE *expression between RMS patients and controls were evaluated using the Mann-Whitney test with Bonferroni's Multiple Comparison correction. Differences between wild type and HPSE silenced cells were compared using the Students' *t*-test. A p value ≤ 0.05 was considered as the level of significance for all tests.

## Results

### HPSE expression in RMS cell lines

**Figure 1:**
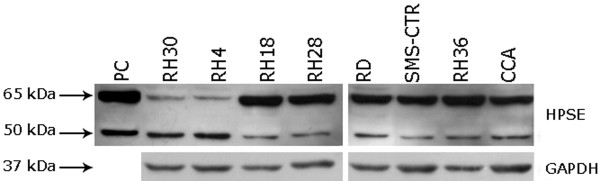
**Heparanase expression in rhabdomyosarcoma cell lines**. Western blot analysis was performed to detect HPSE in total cell lysates. Platelet extract and GAPDH were included as positive and loading controls respectively. One of three independent experiments is reported.

In order to define whether heparanase was expressed in the two major RMS histotypes and whether gene expression correlated with their different metastatic potential, HPSE expression was evaluated in ARMS and ERMS cell lines. Results from absolute quantitative Real-time PCR assay revealed a similar mRNA expression among all cell lines analyzed (data not shown). Subsequently, we performed Western blot analysis to detect HPSE protein in total cell lysates of the same cell lines. Primary polyclonal HPSE antibody was able to recognize both the active (50 kDa) and inactive (65 kDa) isoforms of the enzyme. As shown in Figure 1, both HPSE isoforms were detected in all cell lines under investigation. In particular, ARMS cell lines displayed two different patterns: RH30 and RH4 cell lines were characterized by high levels of the active isoform and low levels of the inactive one, whereas RH18 and RH28 showed a low expression of the active enzyme. Similarly, all ERMS cell lines displayed low levels of active HPSE isoform.

### HPSE activity in RMS cell lines

**Figure 2:**
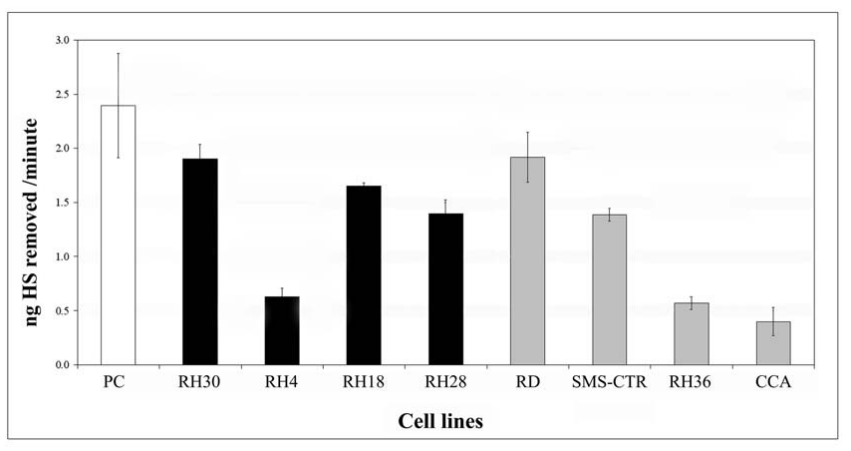
**Heparanase activity in serum-free conditioned media of rhabdomyosarcoma cell lines**. The enzymatic activity is expressed as nanograms of heparan sulphate (HS) removed per minute. The results represent the mean ± standard deviation of three independent experiments performed in duplicate. Platelet extract was used as positive control (PC, white bar); ARMS (black bars); ERMS (grey bars).

HPSE activity in serum-free conditioned media of RMS cell lines was quantified by ELISA assay. The enzymatic activity was expressed as nanograms of HS removed per minute. A marked HPSE activity level could be observed in RH30, RH18 and RH28 as well as in RD and SMS-CTR cells. These cell lines showed enzymatic activity comparable to that of platelets, used as a positive control. Differently, RH4, RH36 and CCA cells displayed a lower enzymatic activity (Figure 2).

### Stable HPSE silencing of RH30 and RD cell lines and invasion assay

**Figure 3:**
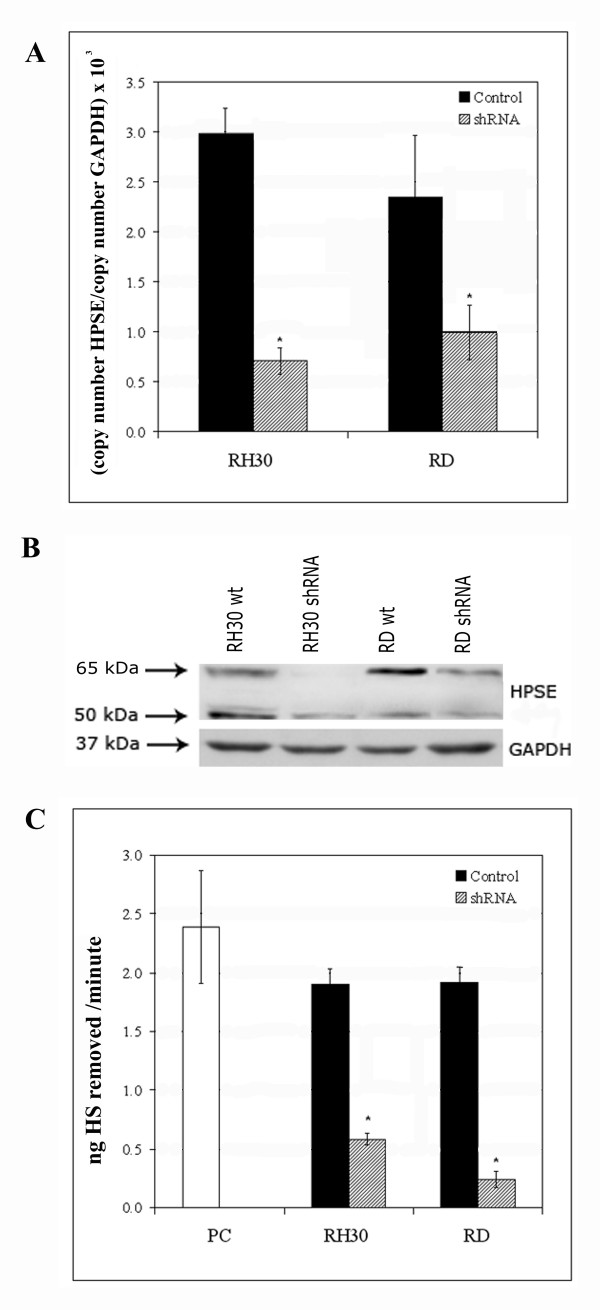
**Stable heparanase silencing of RH30 and RD cell lines with shRNA**. **(A) ***HPSE *mRNA expression levels in control (black bars) and silenced (hatched bars) RH30 and RD cell lines were determined by Real-time PCR. The results were normalized using *GAPDH *as internal control and represent the mean ± standard deviation of three samples performed in duplicate. **(B) **Western blot analysis was performed to demonstrate HPSE silencing between control and silenced RH30 and RD cell lines. GAPDH was included as loading control. **(C) **Heparanase activity in serum-free conditioned media obtained from control (black bars) and silenced (hatched bars) RH30 and RD cell lines. The enzymatic activity is expressed as nanograms of heparan sulphate (HS) removed per minute. The results represent the mean ± standard deviation of three independent experiments performed in duplicate. Platelet extract was used as positive control (PC, white bar). An asterisk (*) indicates a significant difference from control (P < 0.05).

To further investigate the role of *HPSE *expression in RMS, we downregulated *HPSE *in RH30 and RD cell lines. In particular, from each 29 mer-shRNA targeting *HPSE *and the relative negative controls, we obtained three clones under puromycin selection. We evaluated *HPSE *mRNA expression in all clones by Real-time PCR analysis (data not shown) and we chose the pHPSE-3 sequence that showed the best silencing rate in both cell lines. In this way we obtained a significant knockdown of gene expression equal to 76% and 58% for RH30 and RD, respectively (P < 0.05) (Figure 3A). A clear reduction in HPSE protein expression was observed in total cell lysate (Figure 3B). As regards with HPSE activity in serum-free conditioned media from untreated and silenced RH30 and RD cells, we detected a remarkable reduction equal to 70% and 87%, respectively (P < 0.05) (Figure 3C). HPSE expression was comparable in wild type cell lines and in negative control clones (data not shown).

MTT assay did not reveal any differences in cell proliferation between wild type and silenced cells (data not shown).

**Figure 4:**
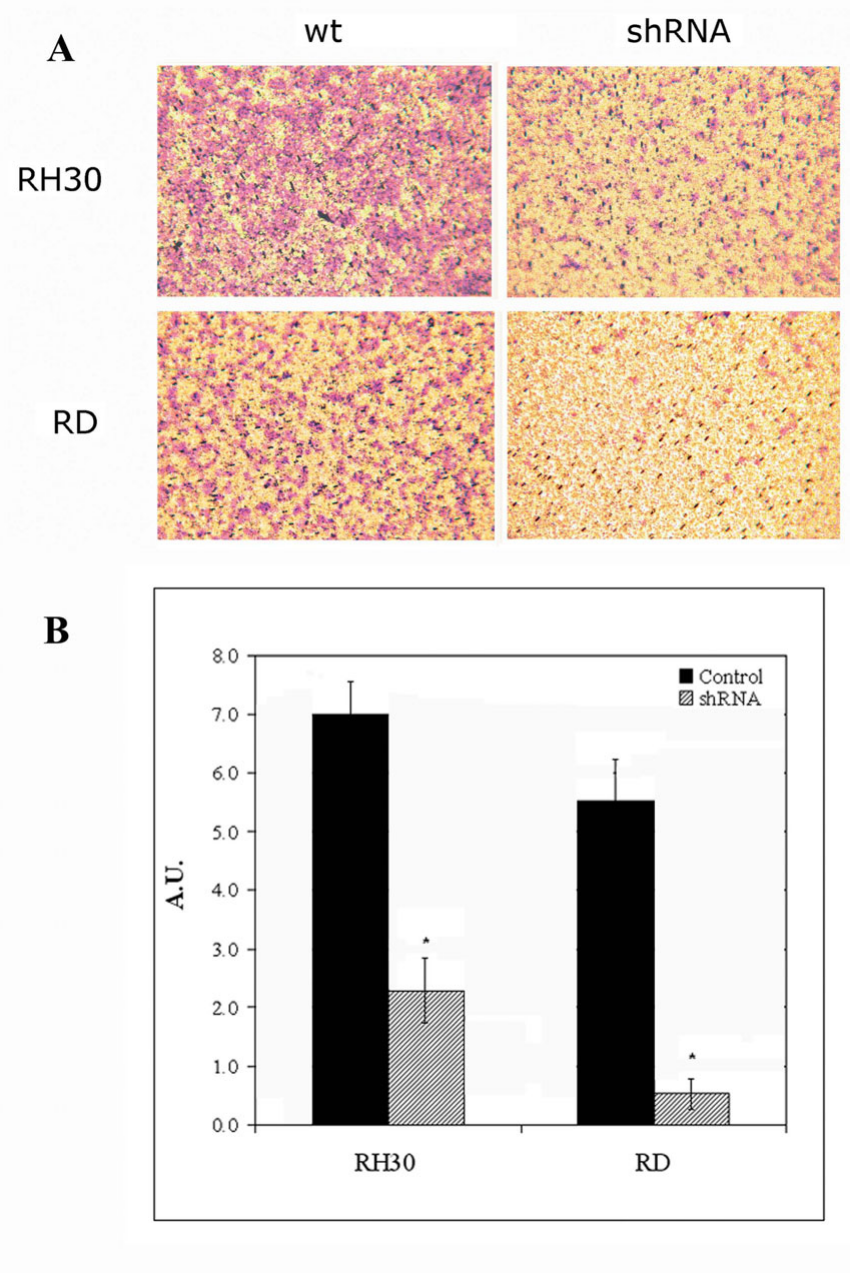
***In vitro *invasion assay**. Invasiveness of RH30 and RD wt (wild type) cells together with RH30 and RD shRNA (silenced) cells was measured using a modified Boyden chamber assay in the presence of NIH/3T3 cell conditioned medium as chemo-attractant. **(A) **One of three independent experiments is reported (magnification 400×). **(B) **The results represent the mean ± standard deviation of quintuplicate samples from three independent experiments. A.U., arbitrary unit. Control corresponds to wild type cells (black bars); shRNA corresponds to *HPSE *silenced cells (hatched bars). An asterisk (*) indicates a significant difference from control (P < 0.05).

Since the ability of malignant cells to invade Matrigel-coated filters represents a measure of their invasiveness, an invasion assay was carried out. We compared RH30 and RD wild type cells with the corresponding silenced cells. As shown in Figure 4, RH30 wild type cells were more invasive than RD wild type cells. As to the role of HPSE in the invasiveness, silenced RH30 and RD cells showed an invasive capacity reduced by 67% and 91% (P < 0.05) of controls, respectively.

### MMPs expression in RH30 and RD cell lines after stable HPSE silencing

In order to define whether *MMPs *expression changes after *HPSE *silencing, we evaluated *MMP2*, *MMP9 *and *MMP14 *expression in RH30 and RD wild type cells and in the corresponding silenced cells by a relative quantitative Real-time PCR.

**Figure 5:**
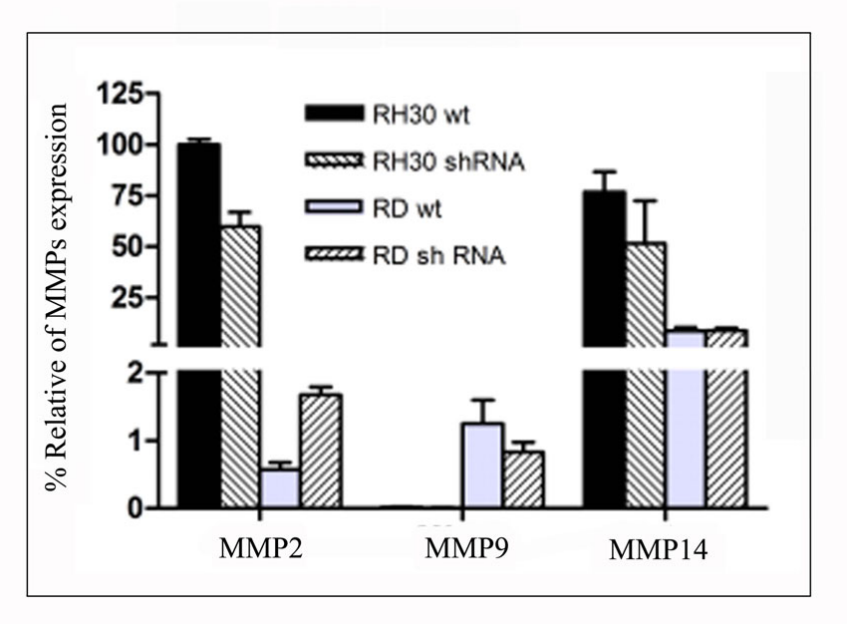
**Matrix Metalloproteinases expression in RH30 and RD cell lines**. *MMPs *mRNA expression levels in control (black and gray bars) and silenced (hatched bars) RH30 and RD cell lines were determined by Real-time PCR. The results were normalized using *GAPDH *as internal control. The expression level determined for *MMP2 *in RH30 wild type cells is regarded as 100% and *MMPs *expression in the other cell lines are presented as percentage relative to it. The results represent the mean ± standard deviation of two independent experiments performed in triplicate.

As shown in Figure 5, RH30 silenced cells displayed a not significant faint decrease of all *MMPs *expression levels with respect to control, whereas in RD wild type and silenced cells *MMPs *level remained substantially equal.

On the whole *MMP2 *and *MMP14 *expression was higher in the alveolar RH30 cells respect to the embryonal RD.

### HPSE expression and activity in RMS patients

*HPSE *expression was evaluated in 12 RMS biopsies by an absolute quantitative Real-time PCR, using normal foetal skeletal muscle cDNA as control.

**Figure 6:**
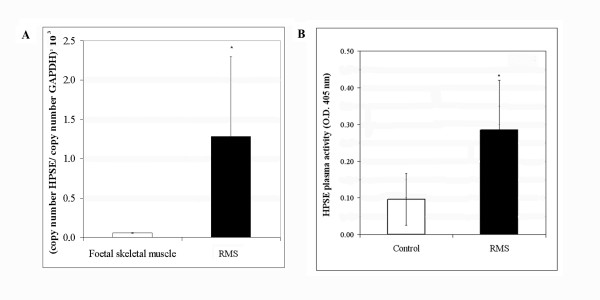
**Heparanase expression and activity in rhabdomyosarcoma patients**. **(A) ***HPSE *mRNA expression levels in foetal skeletal muscle (white bar) and RMS patients (black bar) were determined by Real-time PCR. The results were normalized using *GAPDH *as internal control and represent the mean ± standard deviation of two independent experiments performed in triplicate. **(B) **HPSE activity in each plasma sample was calculated as difference between the O.D. 405 value with or without heparin at the final concentration of 50 μg/ml. An asterisk (*) indicates a significant difference between RMS patients and controls (P < 0.05).

*HPSE *mRNA expression was significantly higher in biopsies of RMS patients compared to foetal skeletal muscle (P < 0.05) (Figure 6A).

HPSE activity in plasma samples collected at diagnosis from 15 RMS patients and from 10 healthy subjects was determined by an ELISA method. As shown in Figure 6B, we demonstrated that HPSE activity was significantly higher in plasma of RMS patients compared to healthy controls (P = 0.001). In addition, the five plasma samples obtained from ARMS patients showed higher activity levels of HPSE than ERMS, although the difference did not reach statistical significance (data not shown).

## Discussion

RMS is classified into two major histological groups: alveolar and embryonal. The rarer ARMS is more aggressive and associated with a significantly worse outcome than ERMS [3]. The more frequent tumor dissemination and metastatic characteristic of ARMS, compared to ERMS, suggest that different enzymatic activities may be involved in host tissue invasion [19]. Whereas upregulation of heparanase is well documented in an increasing number of human solid tumors showing a correlation with their invasive potential [20], its role has not been elucidated in RMS thus far.

Therefore, in the present study, we investigated HPSE expression and activity in RMS cell lines in order to establish whether any correlation between HPSE levels and histotype exists.

HPSE protein may be present both in the active/inactive form. Only the active one (50 kDa) is responsible for HS degradation in the ECM [21]. Since *HPSE *mRNA expression results similar in all RMS cell lines analyzed, we performed a Western blot analysis to discriminate between the two isoforms.

All RMS cell lines showed the expression of HPSE protein. All ERMS cell lines along with RH18 and RH28 showed a similar and moderate expression of active HPSE isoforms and high levels of inactive HPSE isoforms. On the other hand, RH30 and RH4 cell lines were characterized by high levels of active HPSE and moderate expression of the inactive one. It was interesting that the total amount of HPSE protein, the active plus the inactive isoforms, was comparable in all RMS cell lines.

Furthermore, we evaluated HPSE activity by an ELISA assay on serum-free conditioned media from RMS cell lines. This assay confirmed a heterogeneous HPSE activity among RMS cell lines without a clear-cut difference between ARMS and ERMS cell lines.

The difference between active and inactive heparanase isoforms in RMS cell lines lysates and the discrepancy between the cytoplasmic amount of the enzyme and its activity in conditioned media could be explained in light of HPSE complex protein activation and secretion. In fact, heparanase is synthesized as a 65 kDa inactive precursor, shuttled to the Golgi apparatus and then secreted via vesicles. Once secreted, heparanase interacts with HSPGs forming a complex which is endocytosed. Conversion of endosome in lysosome results in HPSE cleavage, yielding 8 and 50 kDa protein subunit that heteodimerize to form the active enzyme [22,23]. Moreover, heparanase active heterodimer can get secreted in response to local or systemic cues [12].

In order to determine the HPSE role in the ECM degradation associated with the different invasive potential in alveolar and embryonal histotypes, we stably silenced one cell line from each histotype by shRNA technique. Based on the ELISA assay results, we selected ARMS RH30 and ERMS RD cell lines characterized by the highest HPSE activity in the conditioned media. After puromycin selection, clones appearing to be silenced more than 50% were chosen from both cell lines. Using a Matrigel-invasion assay, we demonstrated that *HPSE *silencing significantly reduced the invasive potential of RH30 and RD cell lines compared with the untreated cells. Although previous experiments demonstrated a relevant role of other ECM degrading enzymes [19], *HPSE *silencing emphasizes the importance of this enzyme both in alveolar and embryonal RMS invasiveness. Because Zcharia E. *et al*. have recently demonstrated in a knock-out mouse model that absence of *HPSE *expression was associated with an increased expression of *MMPs *[24], we determined the effects of *HPSE *silencing in RH30 and RD cell lines by analyzing the expression of *MMP2*, *MMP9 *and *MMP14*. In our cellular model we did not observe the same phenomenon since *HPSE *silencing did not result in significant differences of *MMPs *expression in silenced vs. wild type cells. In addition, after *HPSE *silencing, no change in cell proliferation was observed by MTT analysis.

It has recently been observed that elevated HPSE levels in patients' blood correlates with a poor prognosis [25]. For this reason we analyzed by an ELISA assay HPSE activity in plasma samples from RMS patients and healthy subjects. RMS patients showed a statistically significant higher heparanase activity with respect to healthy individuals. Coherently, a significantly higher expression of *HPSE *transcripts was assessed in biopsies from RMS patients compared to foetal skeletal muscle tissue used as normal counterpart of the tumor.

Although high HPSE plasma levels have been previously reported in other paediatric malignancies [15], this is the first demonstration of a significantly high HPSE expression in RMS patients. Additionally, although it should be interpreted with caution due to the limited number of patients, there is some suggestion of higher HPSE plasma levels in ARMS compared with ERMS patients. Further clinical and histo-pathological analyses on larger cohorts of RMS patients are necessary to establish whether HPSE may be considered as a novel marker able to distinguish the more aggressive ARMS from ERMS. Since a single functional heparanase has been identified so far [26], the relevant effect of the specific gene knockdown described here should encourage the development of novel heparanase-targeting therapeutic approaches aimed at inhibiting RMS invasive potential.

## Conclusion

In conclusion, we detected for the first time HPSE expression and activity in RMS and showed its implication in tumor cell invasiveness *in vitro*. We also demonstrated a significantly high HPSE expression in RMS patients with a trend to higher levels in ARMS.

## Competing interests

The authors declare that they have no competing interests.

## Authors' contributions

VM carried out immuno-blotting analysis, ELISA assays and HPSE silencing of RMS cell lines; CM and ET carried out qRT-PCR, cell transfections and invasion-assay. AR evaluated the clinical aspects and together with AZ collected plasma and mRNA samples of RMS patients. MO supervised experimental work and wrote the manuscript with the contribution of all co-authors. All authors read and approved the final manuscript.

## Pre-publication history

The pre-publication history for this paper can be accessed here:

http://www.biomedcentral.com/1471-2407/9/304/prepub
